# The Failure of Environmental Education (and How We Can Fix It)

**DOI:** 10.1371/journal.pbio.0050120

**Published:** 2007-04-17

**Authors:** Daniel T Blumstein, Charlie Saylan

## Abstract

The authors argue that environmental education curricula have failed to create an ecologically literate and environmentally sensitive populace, and suggest seven ways that an "evidence-based" approach can help.

On April 22, 1970, one of us (Blumstein) went down to the notoriously foul Schuylkill River and collected a water sample for a display highlighting air and water pollution in Philadelphia's Rittenhouse Square. Blumstein remembers being surprised that polluted water could be clear. On that first Earth Day, pollution was rampant, and environmental literacy was limited in our rapidly urbanizing culture—and efforts to raise awareness met significant resistance.

Environmental education and legislation grew out of a grassroots movement that was generally regarded as counter-culture. The claims and research of early environmentalists, like “Silent Spring” author Rachel Carson, were vigorously attacked and discounted by the chemical industry and their political cronies. Today, attacks on environmentally sound and sustainable policies have largely shifted from the personal to the economic, with critics arguing that sustainable practices would place too high a burden on various economic sectors. Economic hardship is one of the main reasons cited by President George W. Bush for the United States' withdrawal from the Kyoto Protocol in 2001.

While the environmental community's persistence under such adversarial pressure is admirable, we believe that years of constant conflict created a bunker mentality, causing the environmental community to remain exclusively focused on its ideological goals and on self-protection. Even pragmatic evaluations of efficacy were viewed as an assault against the movement, and they were often rejected without acknowledging important criticisms [1,2].

With some trepidation, we would like to argue that it is time for the environmental education community to take stock of itself. Problems lie not only with what has been taught, we believe, but also with the way environmental education curricula have been developed and evaluated. We will challenge the hypothesis that environmental education is successful, and we will suggest ways that an “evidence-based” approach can improve environmental education. Finally, we suggest that to create environmentally aware citizens, some difficult lessons must be taught.

**Figure:**
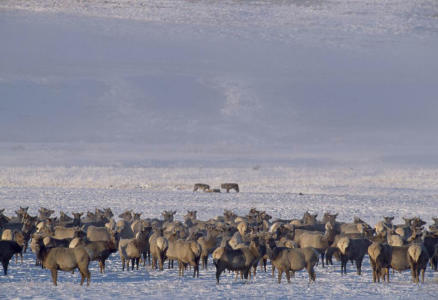
In a demonstration of ecological linkages, reintroducing gray wolves into Yellowstone National Park drove their prey out into the open and away from dense patches of willows along streams, which allowed heavily grazed willows to rebound and provide more habitat for songbirds. (Image: Dan and Cindy Hartman)

We believe that the current level of environmental destruction is unique in the history of humanity, and that if it is left to continue at the current rate, we will, within our generation, experience the initial phases of a trend that seriously risks destroying the very fabric of our lives. We believe that over-consumption lies at the heart of the environmental crisis, that environmental education must teach consumption control, and that ultimately we can help preserve biodiversity and our own environmental welfare by changing our consumption patterns. We believe that if some of our suggestions are adopted, a more ecologically literate and environmentally sensitive populace will follow—and ultimately the environmental problems that threaten our subsistence will be solved.

Since that first Earth Day, substantial resources have been allocated worldwide to environmental education. Most of our examples focus on environmental education in the US, because we are most familiar with these programs and their evaluation. But environmental education occurs throughout the world, in part guided by the international environmental education goals and standards adopted by United Nations Educational, Scientific and Cultural Organization (UNESCO)–sponsored meetings (the 1975 Belgrade Charter [3] and the 1977 Tbilisi Declaration [4]). Addressing the issues that we identify should have global consequences.

In the US, kindergarten–through–12th grade curricula include environmental education modules, and environmental educators who work in the private sector provide highly sought after extra-curricular activities to school districts unable to develop their own programs and to parents who want their children to have more positive environmental experiences. Yet by most objective measures, this money has been wasted, because out of the classroom, people have failed to make the link between their individual actions and the environmental condition. For example, per capita fuel consumption has increased worldwide, with the US leading the pack by a significant margin [5]. Car size has increased notably over the past three decades, with sport utility vehicles and light-duty truck sales, until very recently, making up a large proportion of the passenger vehicle market [6]. Additionally, the fuel economy of modern smaller vehicles has dropped to its lowest point in over two decades [7]. These trends have contributed to a dramatic increase in greenhouse gas emissions since the advent of the industrial age, leading us toward a global climate change of potentially catastrophic proportion and duration [8]. During the past 50 years, between one half and one fifth of all terrestrial biomes capable of producing crops have been modified by human activity [9]. This activity has resulted in a massive loss of ecosystem function and has lead to a serious debate about oil and gas exploration in one of the last large, untouched pieces of Earth—the Arctic National Wildlife Refuge. Ironically, we do not know how much worse it would have been without this investment in environmental education. But the current disconnect between environmental education and personal responsibility is not reassuring and should cause one to stop and reevaluate both the process by which we teach environmental education and the subjects taught.

Some studies have found that although civic scientific literacy in the US has improved since 1970, only about 20% of the public is considered scientifically literate [10]. Despite this modest literacy, we have no good data on the public's awareness or understanding of environmental problems, nor on whether the public has learned what must be done to preserve biodiversity or reduce the threats to our environment. Many people see a hydrogen economy as part of the solution, for example, but they fail to make the link between clean-burning fuel cells and the source of energy required to make those fuel cells. Coal-fired fuel cell plants may concentrate pollution, but they certainly won't eliminate it. And, the recent call by President George W. Bush for ethanol to replace or supplement automobile fuels may not solve the problem, because ethanol production requires land to be cultivated using carbon-based fertilizers and fuel-burning vehicles for planting, harvesting, and transportation. Modern agribusiness relies extensively on fossil fuels [11]. While older ethanol-production technologies used more energy than they produced [12], proponents argue that new technology will be more efficient; a suggestion that remains to be properly evaluated.

Of course, there have been some modest successes. The Endangered Species Act was passed in 1973, and while the act and listings continue to meet resistance from landowners and private interests, some species, such as gray wolves in the lower 48 states, have been rescued from the verge of extinction [13]. We have banned DDT in the US. Pressure on the propellant industry led to the replacement of ozone-depleting CFC propellants, such as Freon. Virtually all tuna is now “dolphin-safe,” a designation that reflects practices designed to reduce the accidental capture of marine mammals. Air and water quality throughout the US has improved (notably in Los Angeles, California, our hometown, where in 1999, there were no smog alerts at all, compared with the 121 first-stage and 11 second-stage smog alerts in 1972 [14]). But these largely legislative victories cannot properly test the effectiveness of environmental education, because they fail to measure directly the impact of environmental education on individual behavior.

More importantly, should such successes truly be considered successes? Consider recycling. We certainly recycle a lot more than we did in 1970, but we still don't know whether recycling really works in a practical and sustainable way. We have successfully concentrated on the mechanics of the recycling process without devoting adequate effort to making sure that the process is economically feasible, and thereby sustainable. This will likely be accomplished by legislative action, governmental incentives and disincentives, and institutional cultivation of “environmental entrepreneuring,” all of which are key factors in developing a place for recycled products in the global marketplace. Viewed this way, recycling should not be called a success until recycled products are routinely used in the manufacturing process. Importantly, recycling a product takes far more energy than reusing it—or simply reducing consumption in the first place.

Consider the Amazon rainforest and the several decades of worldwide efforts to stem the trend of deforestation through education, public awareness, and the implementation of sustainable development strategies. Recent figures released by the Brazilian government show that in the past three years, deforestation levels have averaged above 25,000 square km per year. This represents a failure of environmental protection policy in favor of lucrative soy and cattle farming. We have now lost 20% of the largest tropical forest in the world, an area that contains an estimated 30% of global biodiversity [15]. While some may consider Brazilian development far removed from classroom lessons in North America, US citizens are purchasing both forest and farm products from Brazil—making us partially responsible for Brazil's deforestation. This distressing fact suggests that as environmental educators, we should take a long, hard, and pragmatic look at our methods, because we are failing to make an impact on the problems that are taught in the classroom. Our educational efforts are intended to bring these issues to light and stimulate individual action or increase public awareness, yet they fail to provide the essential support, economic pressure or motivation, or legislative change necessary to create significant, measurable changes on specific issues. Viewed from this perspective, the environmental education community may have failed the greater cause and may indeed have lost sight of its original purpose.

Education involves learning; learning, according to psychologists, is seen when an experience or set of experiences changes behavior. Yet not all changes in behavior can be attributed to learning. For example, older children behave differently than younger children for a variety of reasons, including changes in hormone levels as children age. Thus, psychologists have developed strict protocols to determine what evidence is required to properly demonstrate learning, including trials in which individuals must be “pre-tested,” trained in some way, and then “post-tested.” The objective measure of learning is the difference between post-test and pre-test scores. Importantly, there must also be controls that receive no training; controls allow us to isolate, specifically, the effect of learning. Thus, in the field's jargon, we must use a “before-after, treatment-control” design to properly evaluate learning.

Let's examine a concrete example. Pick two classrooms. Go to both classrooms and survey how many children's' homes recycle. Teach the benefits of recycling to only one of these classes. Survey both classrooms again some weeks after the lessons on recycling. If teaching recycling were effective, then we would expect to see a specific increase in recycling in the class where there was a lesson on recycling, and the increase should be greater than that in the other classroom. Of course, a more scientific approach to doing this experiment would involve larger sample sizes (to be more certain about the outcome), and random assignment of treatments (i.e., the rooms with the recycling lessons) and controls (i.e., the rooms without the recycling lessons). It is difficult to say much about the efficacy of environmental education if only students in higher income neighborhoods are exposed to extra-curricular activities. Thus, the treatments and controls must be randomly assigned with respect to location, socioeconomic status, school quality, etc. Remember, while we claim that environmental education has failed, we have no proper control group with which to properly evaluate how bad things might be if there had been no educational efforts.

Such scientific approaches to evaluate the efficacy of environmental education are either not conducted or are conducted over such a short time period that we have absolutely no idea whether programs or lessons actually work. We are aware of no long-term follow up tests designed to quantify environmental literacy. Moreover, in our experience, while educators feel responsible for providing information or illumination of any given topic, they do not see themselves in the role of changing specific long-term behaviors. Perhaps this feeling stems from environmental education's historical focus on teaching respect of nature. Respecting nature is an essential objective of environmental education, but respect alone has clearly not changed our destructive behavior. Respect and appreciation are the first steps toward developing environmentally aware citizens. We maintain that environmental education must go beyond teaching respect and awareness, and must focus more on changing consumption patterns; it is through changing our consumption patterns that we will have the largest impact on our environment.

Thus, testing procedures, as currently implemented, are likely to be designed to provide an inventory of assimilated information with no metrics to gauge changes in lifestyle or behavior. Comprehension of a subject may be measured by a student's ability to memorize key bits of information, rather than the ability to grasp the concepts from which the bits of information measured are derived. This approach has led to a superficially informed society that is unable to act—and it illustrates the failure of environmental education. Given the importance of proper study design and the successes of evidence-based approaches in other fields, we believe that an evidence-based approach to environmental education will help improve the quality of environmental education.

In Box 1 we list seven ideas that can be taught to students as part of a more holistic environmental education program. We realize that many of the specific things that we advocate go against what some may see as a predisposition to use resources as though they were unlimited. Additionally, we realize that politicians must often adopt a short-term view in order to get re-elected, which may compromise conservation in favor of growth. Nevertheless, we have attempted to create a manifesto for the future of environmental education, because we care not only about its future but also about the future of our planet. However, we believe deeply that simply bumbling around without directed objectives, without creating personal empowerment, and without creating measurable positive impacts is a waste of time and money. We believe that putting the “learning” into environmental education will have tremendous outcomes. Changing the focus of what is taught so that it targets individual behavior and helps us overcome some of our human predispositions may be just what we need to recapture the original vision of the environmental movement—and may help combine respect for the natural world with the will to halt its destruction.

**Box 1: Seven Ways to Improve Environmental Education**

We believe these measures—some designed to help us overcome our wasteful tendencies—will help ensure that kindergarten–through–12th grade environmental education has a measurable impact on the environment.

1. Design environmental education programs that can be properly evaluated, for example, with before-after, treatment-control designs. Such approaches represent a sea change from programs today, and we expect considerable resistance from environmental educators. But the environmental community at large must stop rejecting criticism as negative and must embark on a policy of continuing self-evaluation and assessment.
  To be deemed effective, environmental education and the funding process that supports it must also work backward from specific environmental problems by evaluating the degree of actual impact on a specific issue versus the amount of money and energy spent on public education. We may find that a portion of the money and effort now directed toward education would be better spent on legislation and lobbying.
2. Many environmental issues facing us today are caused by over-consumption—primarily by developed countries. Changing consumption patterns is not generally a targeted outcome of environmental education, but we believe it is one of the most important lessons that must be taught. The magnitude of our impact, as first proposed in 1971 by Paul Ehrlich and John Holdren [16], can be viewed as dependent upon population size, affluence (specifically, per capita economic output), and technology (specifically, the environmental output per unit of economic impact). As countries develop, their environmental footprint may expand, and consumption control may become more important. For instance, the recent rapid growth in China has increased the demand for wood, steel, and fossil fuels throughout the world. Unchecked, such growth is simply unsustainable and will have a profound negative effect on the global environment.
  Thus, we need to radically overhaul curricula to teach the conservation of consumable products. Teaching where and how resources come from—that food, clean water, and energy do not originate from supermarkets, taps, and power points—may be an important first step.
3. We need to teach that nature is filled with nonlinear relationships, which are characterized by “tipping points” (called “phase shifts”): there may be little change in something of interest across a range of values, but above a particular threshold in a causal factor, change is rapid. For instance, ecology, which focuses on understanding the distribution and abundance of life on Earth, is a complex, nonlinear science. If environmental education is linear—in other words, if you teach that recycling one beer bottle will save “*x*” gallons of water—people will not have the foundation to think about linkages or nonlinear relationships.
  The return of wolves to the greater Yellowstone ecosystem, which has had a remarkable number of unanticipated effects, is a compelling demonstration of ecological linkages. Wolves create a landscape of fear, changing the habitat-selection behavior of their prey [17]. Rather than spending time in dense patches of willows by streams, deer, elk, and moose now spend more time in the open, where they can detect predatory wolves at a greater distance. This shift has led to a reduction in grazing pressure on the willows, which have literally exploded and now provide more habitat for songbirds. Thus, the introduction of wolves has had the unintended consequence of increasing songbird diversity in the willows of Yellowstone [18].
  Reconstructing past ecosystems can provide a thoroughly different view than the baseline data that each generation adopts as a basis for environmental policy and legislation. For instance, when European sailors first came to the Caribbean, sea turtles were extremely common. After intensive exploitation, turtle populations and the vital ecological roles they play have never been fully recovered. Without a historical component, these baselines will shift as we ratchet our way to inevitable ecological collapse [19].
4. We need to teach a world view. Americans know little of world history and are geographically illiterate. A 2002 poll of 18–24 year olds in nine western countries, ranked the US next to last in geographic literacy [20]. A greater appreciation of the diversity of cultures and peoples in the world should help us realize the selfish consequences of our consumption. “Not in my backyard” is not a sustainable rallying cry in an interconnected world when we are faced with global climate change. We are too late for “think globally and act locally” to work. And, contrary to the statements of President George W. Bush, the American way of life must become negotiable if it is to be sustainable. We have little trouble suffering security-related inconveniences; we should be willing to accept some inconveniences for the opportunity to live in a sustainable environment.
5. We must teach how governments work and how to effect change within a given socio-political structure. We suspect that many individuals will be offended by the thought that large industries have so much sway on the wording of state and federal legislation. We all suffer from polluted water and greenhouse gasses, but lobbyists are very effective in diluting potentially costly legislation meant to safeguard our water supplies or prevent rampant climate change. Understanding how the system works will empower subsequent generations to change it.
6. We must teach that conservation-minded legislation may deprive us of some of the goods and services that we previously enjoyed. Inexpensive airline flights make flying routine, but planes create more greenhouse gases than trains or buses [21]. Self-sacrifice will be necessary to some degree if we are to avoid or minimize adverse effects of imminent environmental threats with truly global consequences.
7. Finally, we must teach critical thinking. Environmentally aware citizens must be able to evaluate complex information and make decisions about things that we can't currently envision. True scientific literacy means that people have a conceptual tool kit that can be applied to a variety of questions. Unfortunately, much science education is not inspired, and students are required to learn facts without being given the ability to manipulate and analyze those facts. Without the ability to ask questions, identify assumptions, and make well-reasoned decisions, we're left with a population ripe for exploitation by less-than-honest industries and politicians.
